# Comparison of PRISM and numeric scale for self-assessment of learning progress during a clinical course in undergraduate dental students

**DOI:** 10.1186/s12909-022-03967-7

**Published:** 2022-12-24

**Authors:** Gerhard Schmalz, Deborah Kreher, Tom Sensky, Stefan Büchi, Dirk Ziebolz

**Affiliations:** 1 Department of Cariology, Endodontology and Periodontology, University of Leipzig, Leipzig, Germany; 2 Department of Medicine, Centre for Mental Health, Imperial College London, London, UK; 3 Clinic for Psychotherapy and Psychosomatics ‘‘Hohenegg’’, Meilen, Switzerland

**Keywords:** Dental education, PRISM, Visual metaphor, Undergraduate education, Self-reflection, Communication

## Abstract

**Objectives:**

To compare Pictorial Representation of Illness and Self-Measure (PRISM) and a numeric scale for self-reflection in dental students.

**Methods:**

Fourth year dental students were randomly assigned to each receive one interview based on PRISM or a numeric scale to self-assess their competencies at the beginning (t1), the middle (t2) and the end (t3) of integrated clinical course. Questionnaires were used to assess self-perceived benefit of the interviews at each time points.

**Results:**

Students in PRISM group perceived a higher benefit regarding the self-assessment of their practical skills at all time points (*P* < 0.05), for theoretical knowledge at t2 and t3 (*P* < 0.05) and reaching the course objectives at t3 (*P* = 0.04). At all time points, PRISM group rated their interview (*P* = 0.04), the applied instrument (PRISM, *P* = 0.01) and the benefit of the combination of both higher than numeric scale group (*P* < 0.05).

**Conclusion:**

In this preliminary study, PRISM was superior against a numeric scale and can be recommended for dental education to facilitate self-assessment.

**Supplementary Information:**

The online version contains supplementary material available at 10.1186/s12909-022-03967-7.

## Introduction

Dental and medical education are in a phase of transition; the development of student-centered curricula [1], fulfilling the appropriate inclusion of education and training [2], alongside with the increasing demand of the teacher´s role as a coach during medical studies [3, 4] are several issues of importance. For this reason, students’ abilities in self-reflection and self-assessment during their studies are of increasing relevance [5, 6]. Originally, self-reflection means the view of a person by his-/herself, and reflecting on (and learning based on) experiences [7, 8]. In context of education, it means that students reflect on their own strengths and deficiencies, leading to a definition of learning aims and, ideally, learning progress [9]. It has been documented that the ability to self-reflect is associated with academic performance and learning effectiveness [10, 11]. Therefore, self-reflection appears crucial for lifelong learning, especially due to the ability to set goals and evaluate their attainment (self-feedback), helping to learn and to motivate for future tasks [12].

While several approaches are available for self-reflection, a gold-standard or most favorable measure is still missing [13]. In this respect, any measure to support or foster self-reflection is challenged by contemporary tasks like student-centered education or appropriate inclusion of competency-based education [1] and on the other hand by complex issues like the student–teacher relationship [14]. Supporting students to develop self-reflection skills is highly important for contemporary medical education [12]. Considering the high relevance of this topic on the one hand, and the absence of a gold-standard method on the other, there appears a gap in research in the field of dental (and overall medical) education.

Therefore, novel and innovative intervention strategies to support self-reflection in dental and medical education appear needed and are a potentially promising target of dental education research. Recently, a novel instrument has been introduced in dental education context, which originated from the field of psychology/psychosomatics, i.e., the Pictorial Representation of Illness and Self-Measure (PRISM) [15]. PRISM is a visual metaphor, which was primarily developed to measure suffering, especially helping patients with severe chronic general diseases [16, 17]. In a modified form, PRISM has been applied to undergraduate dental education, whereby the context of the task was transferred into dental studies. Previous studies used PRISM for self-reflection in the field of conservative dentistry and periodontology, showing that students perceived a benefit of the visual metaphor, which was also experienced to support the student–teacher-relationship [18]. Moreover, PRISM was also sensitive as a quantitative measurement of subjectively perceived gain in competencies during a simulation course in conservative dentistry [19]. Although those previous studies showed that PRISM is a promising tool to foster self-reflection in dental education, it has not been tested against other measures, yet. Based on a recent systematic review, rubric-tools, e.g. numeric scales are the most commonly used measures for self-reflection [13] and thus might be considered as a kind of reference standard in this context. Many institutes use their own numeric scales for evaluation and self-assessment of the students.

Accordingly, this current study aimed to compare PRISM with a numeric scale as self-reflection tool during a clinical course for undergraduate dental students. Two randomly assigned groups received either three PRISM tasks or numeric-scale based interviews about their competencies (including skills and knowledge, need for education and their perceived distance from reaching the course objectives) during a clinical integrated course. The applied numeric scale was in line with similar rubric tools, but an individually composed, study-specific evaluation instrument. It was hypothesized that students perceive a higher benefit with regard to their self-reflection of PRISM compared to the numeric scale.

## Methods

### Study design

The study protocol was reviewed and approved by the ethics committee of the medical faculty of University of Leipzig, Germany (No: 117/20-ek). This study compared two randomly assigned groups using either the PRISM method (group A) or a numeric scale (group B) for interviews during one term of their clinical course. All participants were informed verbally and in writing and provided their written informed consent.

### Participants and groups

Sample size calculation: a difference in mean of 1.5 points with a standard deviation of 2 should be detected and revealed with a power of 80% and a type error rate of α = 5%. Therefore, a sample size of 17 was necessary. Accordingly, to compensate a potential drop out during follow-up, 18 4^th^ year undergraduate students were recruited to each group (group A / B). Inclusion criteria were starting the first term (winter term 2021/22) in the clinical integrated course on conservative dentistry and prosthodontics as well as consent for voluntary participation. Moreover, students who had already used the PRISM task were excluded from the study. The participating students were randomly assigned to one out of two groups (A/B) by the drawing of lots: group A received three PRISM task-based interviews and group B received three numeric-scale-based interviews during the first part of the clinical integrated course in winter term 2021/22.

### The PRISM task and interview

**Fig. 1:**
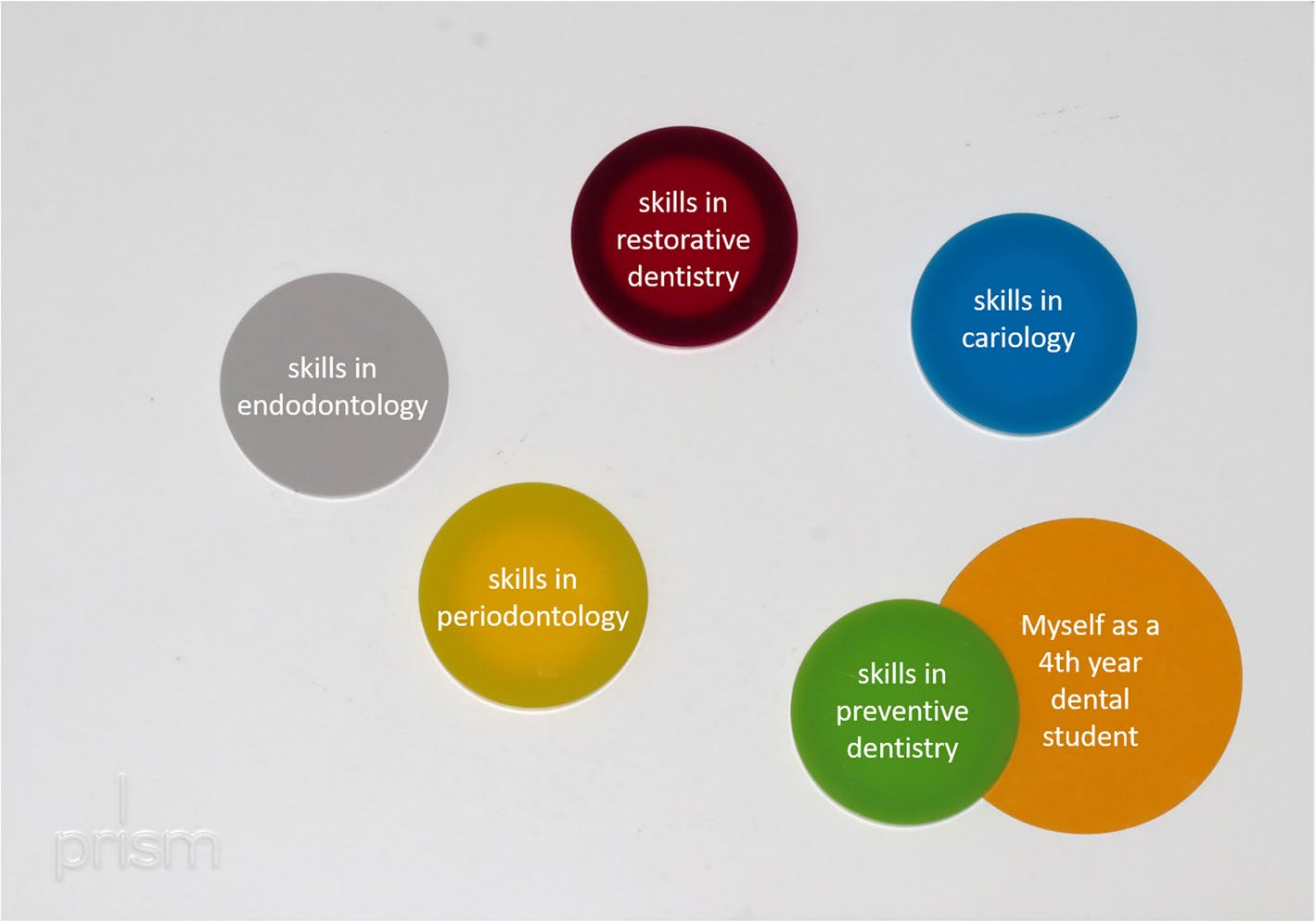
Principle of PRISM interviews in the current study. Each disc represented a sub-aspect of the subject group restorative dentistry and periodontology. The circle in the bottom right hand represents “Myself as a 4^th^ year dental student”, whereby the center of the circle is the “optimum”, i.e. the highest level of skills in the respective field

PRISM is a visual metaphor, which was developed in the field of psychology/psychosomatic medicine [16]. PRISM is able to visualize a relationship between a subject and associated objects in a defined context [17]. The methodic approach consists of a white metal board (210 × 297 mm, “context”), which was defined in the current study to be “Your dental studies”. In the bottom right hand corner of the board, a fixed yellow circle (d = 7 cm) represents the “*Subject”* (“myself as a 4th-year dental student”). Differently colored magnetic discs (d = 5 cm) represent the *“Objects”*, which were different aspects of dental studies like “your practical skills in periodontology”, or “your theoretical knowledge of conservative dentistry” (Fig. 1). The method has already been applied to the context of dental education and was used accordingly [18, 19]. In brief, students were simply instructed to place each “*Object* “ disc, whereby the closer the *“Object”* was placed to the *“Subject”*, the more salient the participant appraises the “*Object*” to be to the “*Subject*” in the defined Context [17]. Accordingly, placing an object close to “myself as 4th-year dental student” reflects a good learning progress.

The PRISM interview consisted of five categories: theoretical knowledge, practical skills, interests, remaining education need and perceived distance to reaching the objectives of the clinical course. In each of those categories, five object disks were placed, reflecting the sub-fields of conservative dentistry (see Fig. 1). Accordingly, 25 singular tasks were solved by the students and discussed with the interviewing teacher (interview time 10–15 min).

### The numeric scale and interview

The numeric scale was developed, consisting of the same questions and issues as the PRISM interview. Therefore, 25 questions, which were in line with the PRISM task were answered on a scale between 0 = very bad/very high and 10 = very good/very high. Students were asked to rate their competencies based on these numeric scales and were able to discuss the results with the interviewer (interview time 8–12 min).

### Scales to evaluate use of PRISM and numeric scale

Two different questionnaires were developed: The first questionnaire (questionnaire A) was devised to assess the subjectively perceived self-reflection abilities of the students. For this, students needed to rate their skills in self-reflection of their own competencies on a scale between 0 = very bad and 10 = very good. The second questionnaire (questionnaire B) was devised to evaluate the perceived benefit of the respective interviews (either PRISM or numeric scale-based). This questionnaire also used a scale between 0 = not helpful and 10 = very helpful. All of the questionnaires used underwent a short pre-test with selected dental students who were not part of the current study to ensure understandability and clarity.

### Study flow

**Fig. 2:**
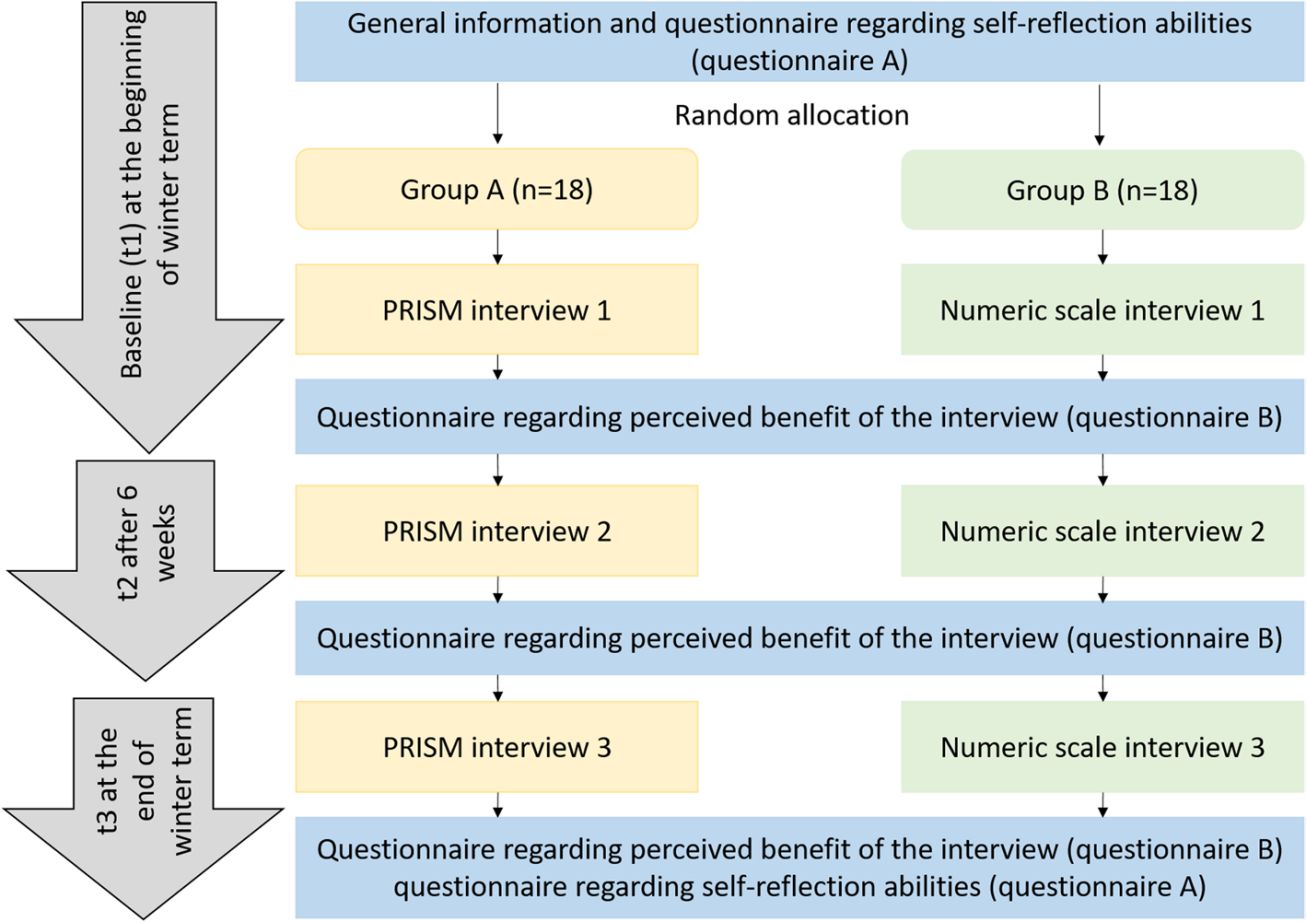
Study flow of the comparison between PRISM and numeric scale

The study flow is shown in Fig. 2. At baseline, all participants received questionnaire A and were allocated to the respective group. Participants received an interview with the respective method (either PRISM or numeric scale) at the beginning of winter term (t1), after six weeks (t2) and at the end of winter term (t3). After each interview, questionnaire B was completed. Finally, at t3, questionnaire A was completed again. Every interview was performed by the same experienced interviewer, who was not involved in the regular course of the students.

### Statistical analysis

The statistical analysis has been performed with SPSS for Windows, version 24.0 (SPSS Inc., U.S.A.). Non-normally distributed data were compared using Mann–Whitney-U-test or by Wilcoxon-test, respectively. Categorical data were analyzed by Fisher exact test. The significance level was set at *P* < 0.05.

## Results

### Questionnaire A shows comparable results between groups

At baseline, the subjectively perceived abilities of self-reflection were comparable in both groups (*P* > 0.05, Table 1). Comparing the results of questionnaire A between t1 and t3, there was neither a statistical significant difference within group A (PRISM group, *P* > 0.05), nor in group B (numeric scale group, *P* > 0.05; Table 2).

**Table 1:** Participants characteristics and baseline results of questionnaire A between groups

	***Group A (PRISM, n* = *18)***	***Group B (numeric scale, n* = *18)***	***P-value***
*Age (mv* ± *sd)*	23.06 ± 2.57	23.38 ± 3.65	0.51^*^
*Gender (% male)*	33%	41%	0.67^**^
*How good is your ability to self-assess the following issues? (0* = *very bad– 10* = *very good)*			
* Theoretical knowledge (mv* ± *sd)*	6.69 ± 1.54	6.75 ± 1.34	0.80^*^
* Practical skills (mv* ± *sd)*	6.13 ± 1.63	6.44 ± 1.41	0.41^*^
* Interests (mv* ± *sd)*	8.31 ± 1.35	8.19 ± 1.38	0.77^*^
* Further need of education (mv* ± *sd)*	7.63 ± 1.50	7.31 ± 1.49	0.64^*^
* Reaching the course objectives (mv* ± *sd)*	7.50 ± 1.86	6.31 ± 2.06	0.09^*^
* How good is your general ability for self- reflection (0* = *very bad – 10* = *very good) (mv* ± *sd)*	6.94 ± 1.57	7.50 ± 1.26	0.23^*^
*How much do you agree with the following statements (0* = *not at all – 10* = *completely)?*			
* Continuous self-reflection is essential for further development of skills and knowledge (mv* ± *sd)*	8.44 ± 0.96	9.13 ± 0.96	0.05^*^
* Ability to self-reflect is essential for a good dentist (mv* ± *sd)*	8.81 ± 1.64	9.19 ± 1.17	0.53^*^
* Self-reflection is important to detect my own interests (mv* ± *sd)*	8.19 ± 1.64	8.69 ± 1.25	0.44^*^
* Self-reflection is important to detect my strengths and weaknesses (mv* ± *sd)*	8.44 ± 1.50	8.87 ± 1.31	0.43^*^

*Mv* mean value, *sd* standard deviation

^*^Mann–Whitney-U test

^**^Fisher test

**Table 2:** Comparison of questionnaire A results between t1 and t3 within groups

	***Group A (PRISM, n* = *18)***			***Group B (Numeric scale, n* = *18)***		
	**T1**	**T3**	***P-value***	**T1**	**T3**	***P-value***
*How good is your ability to self-assess the following issues? (0* = *very bad– 10* = *very good)*						
* Theoretical knowledge (mv* ± *sd)*	6.69 ± 1.54	7.06 ± 1.24	0.40^*^	6.75 ± 1.34	7.37 ± 1.41	0.26^*^
* Practical skills (mv* ± *sd)*	6.13 ± 1.63	6.62 ± 1.75	0.24^*^	6.44 ± 1.41	6.87 ± 1.71	0.46^*^
* Interests (mv* ± *sd)*	8.31 ± 1.35	8.00 ± 1.26	0.30^*^	8.19 ± 1.38	8.31 ± 1.74	0.72^*^
* Further need of education (mv* ± *sd)*	7.63 ± 1.50	7.00 ± 0.89	0.11^*^	7.31 ± 1.49	6.81 ± 1.47	0.34^*^
* Reaching the course objectives (mv* ± *sd)*	7.50 ± 1.86	7.38 ± 1.02	0.80^*^	7.31 ± 2.06	7.63 ± 1.82	0.08^*^
* How good is your general ability for self-reflection (0* = *very bad – 10* = *very good) (mv* ± *sd)*	6.94 ± 1.57	7.38 ± 1.45	0.16^*^	7.50 ± 1.26	7.50 ± 0.89	0.87^*^
*How much do you agree with the following statements (0* = *not at all – 10* = *completely)?*						
* Continuous self-reflection is essential for further development of skills and knowledge (mv* ± *sd)*	8.44 ± 0.96	8.69 ± 1.01	0.38^*^	9.13 ± 0.96	9.00 ± 0.97	0.58^*^
* Ability to self-reflect is essential for a good dentist (mv* ± *sd)*	8.81 ± 1.64	8.44 ± 1.15	0.15^*^	9.19 ± 1.17	8.63 ± 0.89	0.06^*^
* Self-reflection is important to detect my own interests (mv* ± *sd)*	8.19 ± 1.64	8.19 ± 1.05	0.72^*^	8.69 ± 1.25	8.81 ± 1.05	0.56^*^
* Self-reflection is important to detect my strengths and weaknesses (mv* ± *sd)*	8.44 ± 1.50	8.44 ± 1.03	0.97^*^	8.87 ± 1.31	8.75 ± 0.93	0.60^*^

*Mv* mean value, *sd* standard deviation

^*^Wilcoxon test

### Questionnaire B at t1after the first interview shows better results in PRISM group

After the first interview, students in group A perceived a greater benefit of the interview regarding the self-assessment of their practical skills than group B (*P* = 0.04). Furthermore, group A rated their interview (*P* = 0.04) and the applied instrument (PRISM, *P* = 0.02) as more suitable for self-reflection than group B (numeric scale). Additionally, group A rated the benefit of the combination of interview and instrument greater than group B (*P* = 0.03; Table 3).

**Table 3:** Comparison of results of questionnaire B between group A und group B at t1 after the first interview

	***Group A (PRISM, n* = *18)***	***Group B (numeric scale, n* = *18)***	***P-value***
*The interview was helpful for me to self-reflect my competencies regarding the following issues (0* = *not helpful – 10* = *very helpful)*			
* Theoretical knowledge (mv* ± *sd)*	7.38 ± 1.63	6.88 ± 1.86	0.49^*^
* Practical skills (mv* ± *sd)*	7.69 ± 1.54	6.50 ± 1.79	0.04^*^
* Interests (mv* ± *sd)*	7.31 ± 1.49	6.69 ± 2.60	0.70^*^
* Further need of education (mv* ± *sd)*	8.06 ± 1.84	7.31 ± 1.58	0.14^*^
* Reaching the course objectives (mv* ± *sd)*	7.31 ± 1.70	6.63 ± 1.86	0.22^*^
* Will you draw personal consequences from the interview? (%)*	93.3	75.0	0.33^**^
*How much do you agree with the following statements (0* = *not at all – 10* = *completely)?*			
* The interview was appropriate for self-reflection (mv* ± *sd)*	7.81 ± 1.52	6.50 ± 1.86	0.04^*^
* The used instrument is appropriate for self-reflection (mv* ± *sd)*	8.19 ± 1.28	6.56 ± 2.19	0.02^*^
*How high do you rate (0* = *very low – 10* = *very high)?*			
*… the benefit of the combination of interview and instrument (mv* ± *sd)*	8.50 ± 1.10	7.31 ± 1.70	0.03^*^

*Mv* mean value, *sd* standard deviation

^*^Mann–Whitney-U test

^**^Fisher test, significant results (significance level *P* < 0.05) are highlighted in bold

### Questionnaire B at t2 after the second interview confirms better results in PRISM group

After the second interview (t2), students in group A rated their interview more helpful than group B for the self-reflection regarding theoretical knowledge (*P* = 0.01), practical skills (*P* = 0.01) and reaching the course objectives (*P* = 0.04). As at t1, group A rated their interview (*P* = 0.04), the applied instrument (PRISM, *P* = 0.01) and the benefit of the combination of interview and instrument higher than group B (*P* = 0.02; Table 4).

**Table 4:** Comparison of results of questionnaire B between group A und group B at t2 (after 6 weeks, second interview)

	***Group A (PRISM, n* = *18)***	***Group B (numeric scale, n* = *18)***	***P-value***
*The interview was helpful for me to self-reflect my competencies regarding the following issues (0* = *not helpful – 10* = *very helpful)*			
* Theoretical knowledge (mv* ± *sd)*	7.94 ± 1.18	6.75 ± 1.63	0.01^*^
* Practical skills (mv* ± *sd)*	8.25 ± 1.00	6.38 ± 1.96	0.01^*^
* Interests (mv* ± *sd)*	8.13 ± 1.31	7.12 ± 1.75	0.07^*^
* Further need of education (mv* ± *sd)*	7.87 ± 1.50	7.56 ± 1.41	0.43^*^
* Reaching the course objectives (mv* ± *sd)*	8.13 ± 1.02	6.44 ± 2.25	0.04^*^
* Will you draw personal consequences from the interview? (%)*	93.8	75.0	0.33^**^
*How much do you agree with the following statements (0* = *not at all – 10* = *completely)?*			
* The interview was appropriate for self-reflection (mv* ± *sd)*	8.19 ± 1.22	6.81 ± 1.97	0.04^*^
* The used instrument is appropriate for self-reflection (mv* ± *sd)*	8.25 ± 1.06	6.63 ± 2.03	0.01^*^
*How high do you rate (0* = *very low – 10* = *very high)?*			
*… the benefit of the combination of interview and instrument (mv* ± *sd)*	8.69 ± 1.08	7.37 ± 1.86	0.02^*^

*Mv* mean value, *sd* standard deviation

^*^Mann–Whitney-U test

^**^Fisher test, significant results (significance level *P* < 0.05) are highlighted in bold

### Questionnaire B at t3 after the third interview confirms again better results in PRISM group

**Fig. 3:**
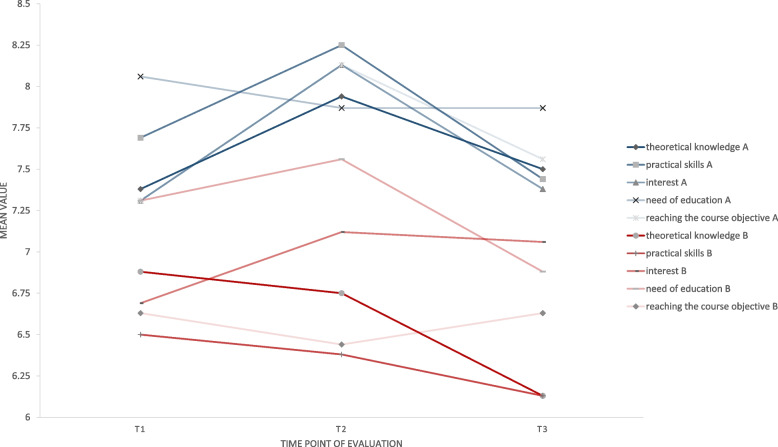
subjectively perceived benefit of the interview (PRISM or numeric scale) with regard to the students’ competencies over the study period (t1-t3). Values for group A (PRISM) are shown as blue lines, while red lines represent the values of group B (numeric scale)

After the third interview (t3), compared with students in group B, those in group A perceived a greater benefit of the interview with regard to the assessment of their theoretical knowledge (*P* = 0.02) and practical skills (*P* = 0.04). As found for the two other time points, group A rated their interview (*P* = 0.04), the applied instrument (PRISM, *P* = 0.01) and the benefit of the combination of interview and instrument more highly than group B (*P* = 0.01; Table 5). The change over time, depending on group and time point is illustrated in Fig. 3 and Supplementary Fig. 1 for visualization.

**Table 5:** Comparison of results of questionnaire B between group A und group B at t3 (end of winter term, third interview)

	***Group A(PRISM, n* = *18)***	***Group B(numeric scale, n* = *18)***	***P-value***
*The interview was helpful for me to self-reflect my competencies regarding the following issues (0* = *not helpful – 10* = *very helpful)*			
* Theoretical knowledge (mv* ± *sd)*	7.50 ± 1.15	6.13 ± 1.86	0.02^*^
* Practical skills (mv* ± *sd)*	7.44 ± 1.31	6.13 ± 2.16	0.04^*^
* Interests (mv* ± *sd)*	7.38 ± 1.86	7.06 ± 1.57	0.50^*^
* Further need of education (mv* ± *sd)*	7.87 ± 1.02	6.88 ± 1.93	0.11^*^
* Reaching the course objectives (mv* ± *sd)*	7.56 ± 1.26	6.63 ± 2.31	0.34^*^
*Will you draw personal consequences from the interview? (%)*	87.5	75.0	0.65^**^
*How much do you agree with the following statements (0* = *not at all – 10* = *completely)?*			
* The interview was appropriate for self-reflection (mv* ± *sd)*	8.06 ± 0.68	7.00 ± 1.59	0.04^*^
* The used instrument is appropriate for self-reflection (mv* ± *sd)*	8.37 ± 0.89	6.44 ± 2.10	0.01^*^
*How high do you rate (0* = *very low – 10* = *very high)?*			
*… the benefit of the combination of interview and instrument (mv* ± *sd)*	8.69 ± 0.70	7.25 ± 1.57	0.01^*^

*Mv* mean value, *sd* standard deviation

^*^Mann–Whitney-U test

^**^Fisher test, significant results (significance level *P* < 0.05) are highlighted in bold

## Discussion

In this study, PRISM was found to be superior to the numerical scale with regard to the subjectively perceived benefit of the interview. This was rather obvious for student´s self-reflection on practical skills and theoretical knowledge than for the other issues. The perceived benefit was greatest at the middle of the course (t2). Moreover, at each time point, students rated PRISM more highly than the numerical scale as an instrument for self-reflection.

In general, self-reflection and critical reflection is important as it supports learning progress throughout one’s studies and is a mandatory ability for the whole working life as a dentist [20, 21]. However, it is a challenge for both students and teachers because reflection is a complex issue, including the processes of analyzing, questioning and reframing of an experience [22]. An appropriate method and a continuity of reflection during a longitudinal curriculum are needed for successful teaching of reflection [22]. As a visual metaphor, PRISM clearly differs from other approaches to foster self-reflection in the dental education setting; several recent examples include e-portfolios, briefing and debriefing sessions before and after clinical practice, reflective writing as well as video-based peer-feedback [23–26]. All of those approaches can be successful and the appropriate instruments and strategy for self-reflection depend on several factors related to the course, practice context and the individual [27]. The time point of using self-reflection is therefore an issue of interest, where usage before, during and after the course is possible, with different advantages and drawbacks, as already described in literature [27].

This current study showed that there was a difference in perceived benefit of the interview, depending on the time point. As illustrated in Fig. 3, the majority of issues had a peak in the middle of the term/course, especially in PRISM group. It is known that the visual metaphor in PRISM task leads to a reflection process whereby a repetition of the PRISM task is favorable [17]. It is therefore not surprising that students experienced a higher benefit in the second interview, when using PRISM. Moreover, the middle of the term might be an appropriate time point to reflect the clinical experience so far and to draw consequences for the rest of the course. This might be less intensive at the first interview (beginning of term) as there was very little clinical experience (first clinical course in conservative dentistry) and at the end of the term as the course was finished at that time. However, as can be seen from Table 4–6, at all time points, the vast majority of students, especially in PRISM group, stated that they were able to draw clinical consequences from the interview. Altogether, continuous usage of a self-reflection instrument during the course appears reasonable, with the greatest importance of an appraisal in the middle of the course.

The main focus of this current study was the comparison between PRISM and a numeric scale. The used scoring between 0 and 10 is as a rubric tool which cannot be seen as a gold standard for self-reflection but a recent review article did not confirm a superiority of any other instrument [13]. Another reason why the numeric scale was applied as control in the current study was because students were quite familiar with this form of appraisal as they knew similar evaluations from their previous studies. In contrast to the quite general approach of a numeric scale, PRISM enables a focus on personally salient information via a visual metaphor [18]. PRISM can therefore foster students’ critical reflection of the own view on different issues, what is one important facet of reflection [28]. A metaphor always requires a distinctly personal interpretation and understanding [17, 29]. This contrasts with use of a generic (and impersonal) numeric scale, which the student can answer without necessarily requiring critical reflection of personal experiences. This offers an explanation for the perceived benefits of PRISM. Furthermore, these findings are in line with the previously highlighted benefits of PRISM in dental education: support of relationship building between teacher and student and the fostering of student's capacity to appraise his/her learning from different perspectives [18]. Therefore, the current study confirmed that PRISM has a self-perceived benefit for the students and can be recommended as a tool to facilitate self-reflection in undergraduate dental students. However, the effect was limited to the interview itself; as can be seen from Table 3, the perceived self-reflection abilities between t1 and t3 did not improved significantly in one of the groups. Although this is limited by the comparably high values at t3, a benefit of PRISM with regard to self-reflection abilities is still not completely evident.

In summary, two main recommendations for the usage of PRISM in such dental educational settings can be provided: first, PRISM should be used in a standardized setting in an interview form. An interviewer with experience in using this method should explain and introduce the task, as described previously [18]. Secondly, based on the current study´s findings, PRISM should be used repeatedly, because students perceive a greater effect with repetitive use of the PRISM task. Completing the PRISM task at the beginning and again at the middle of a course appease to be most effective. Used thus, PRISM can foster self-reflection and help students to develop their competencies.

## Strengths and limitations

Strengths of this study included that it tested a novel method to assess self-reflection, that students were randomized into the two groups tested, that the groups were comparable at baseline, and that the sample size was sufficient, based on a power calculation. Nevertheless, despite meeting power calculation requirements, the groups were overall small, and the findings require interpretation with caution. That the students were all taking a single course lasting an academic term was both an advantage and a disadvantage. Because the students were participating in the same course, comparisons between the two groups were valid. However, a longer observation time, perhaps incorporating a cross-over design, would have strengthened the conclusions from the results. For this reason, the results of the present study should be regarded as preliminary. The questionnaires were specifically designed for use in the study. This had the advantage that the questions reflected those covered in the PRISM task, but the questionnaires were subjected to only basic validation. Because questionnaire B was applied for the first time after a first intervention, it is unclear, whether the participants would have shown any significant differences before the study started. The interviews were performed by a very experienced teacher, as PRISM is quite technique sensitive; this limits the generalizability and transferability of the current findings. Regarding the overall methodology of the current study, another limitation requires consideration; it is unclear whether the current quantitative approach used in the study could adequately capture the true value of using PRISM, as the tool itself is intended to be a representation of self-reflection of the learners after certain learning activities. Accordingly, a more qualitative approach to investigate the usefulness of the tool, compared to that of the numeric scale would be reasonable and valuable. In a previous validation, PRISM was discussed in a focus group with students, showing several benefits and limitations of the method [18]. Similarly, a qualitative assessment of the value of PRISM during a clinical course in dental education is recommendable for future studies in the field. While not relevant to the design of the study, it should also be noted that the PRISM task needs to be set up with care [17] and optimal use most likely benefits from training.

## Conclusion

In this preliminary study, PRISM, a visual metaphor instrument, was rated as more beneficial than a numeric scale for self-reflection of different competencies (especially practical skills and theoretical knowledge) among dental students. PRISM can therefore be recommended for application in dental education settings to facilitate self-assessment of learning progress. Best results are likely if PRISM can be used repeatedly during a course.

## Supplementary Information

**Additional file 1:** **Supplementaryfigure 1.  **Perceived benefit of interview, instrument and the combination of both depending on group and time point. The y-axis shows the mean values of the respective issue.

## Data Availability

The datasets used and/or analyzed during the current study are available from the corresponding author on reasonable request. The data are not publically available, because of the psedonymisation and data protection guidelines according to the ethics approval.

## References

[1] Chuenjitwongsa S, Oliver RG, Bullock AD (2018). Competence, competency-based education, and undergraduate dental education: a discussion paper. Eur J Dent Educ.

[2] Bateman H, Stewart J, McCracken G, Ellis J (2021). Undergraduate dental education: an education or training?. Br Dent J.

[3] Lovell B (2018). What do we know about coaching in medical education? a literature review. Med Educ.

[4] Wolff M, Morgan H, Jackson J, Skye E, Hammoud M, Ross PT (2020). Academic coaching: Insights from the medical student's perspective. Med Teach.

[5] Krupp MM, Barlow PB, Kyle EJ (2021). Developing a self-assessment tool for dental faculty to map professional growth. J Dent Educ.

[6] Tricio JA, Woolford MJ, Escudier MP. Fostering dental students’ academic achievements and reflection skills through clinical peer assessment and feedback. J Dent Educ. 2016;80(8):914–23 (PMID: 27480702). 27480702

[7] Bürgy M. Selbstreflexion, Interpersonalität und psychoanalytische Ethik [Self-reflection, interpersonal behavior and psychoanalytic ethics]. Psychother Psychosom Med Psychol. 1997;47(5):181–6. German. PMID: 9265199. 9265199

[8] Gianakos D. Self-reflection, learning, and sharing mistakes. Pharos Alpha Omega Alpha Honor Med Soc. 1999;62(4):33–4 (PMID: 10992920). 10992920

[9] Richards JB, Hayes MM, Schwartzstein RM (2020). Teaching clinical reasoning and critical thinking: from cognitive theory to practical application. Chest.

[10] Lew MD, Schmidt HG (2011). Self-reflection and academic performance: is there a relationship?. Adv Health Sci Educ Theory Pract.

[11] Pai HC, Ko HL, Eng CJ, Yen WJ (2017). The mediating effect of self-reflection and learning effectiveness on clinical nursing performance in nursing students: a follow-up study. J Prof Nurs.

[12] Deiorio NM, Carney PA, Kahl LE, Bonura EM, Juve AM (2016). Coaching: a new model for academic and career achievement. Med Educ Online.

[13] Williams JC, Ireland T, Warman S (2019). Instruments to measure the ability to self-reflect: a systematic review of evidence from workplace and educational settings including health care. Eur J Dent Educ.

[14] Gillespie M (2005). Student-teacher connection: a place of possibility. J Adv Nurs.

[15] Büchi S, Sensky T, Sharpe L, Timberlake N (1998). Graphic representation of illness: a novel method of measuring patients' perceptions of the impact of illness. Psychother Psychosom.

[16] Büchi S, Buddeberg C, Klaghofer R (2002). Preliminary validation of PRISM (Pictorial Representation of Illness and Self Measure)–A brief method to assess suffering. Psychother Psychosom.

[17] Sensky T, Büchi S (2016). PRISM, a novel visual metaphor measuring personally salient appraisals, attitudes and decision-making: qualitative evidence synthesis. PLoS ONE.

[18] Schmalz G, Sensky T, Kullmann H, Büchi S, Ziebolz D. PRISM – a novel visual instrument to facilitate self-reflection and learning progress. BioMed Research International, vol. 2022, Article ID 2009894, 12 pages, 2022. 10.1155/2022/2009894 10.1155/2022/2009894 PMC9398765 36017381

[19] Schmalz G, Kullmann H, Sensky T, Kreher D, Haak R, Büchi S, Ziebolz D (2022). Pilot study to evaluate a novel measure of self-perceived competencies among dental students. BMC Med Educ.

[20] Sandars J. The use of reflection in medical education: AMEE Guide No. 44. Med Teach. 2009;31(8):685–95. 10.1080/01421590903050374 19811204

[21] Tagawa M, Imanaka H (2010). Reflection and self-directed and group learning improve OSCE scores. Clin Teach.

[22] Aronson L (2011). Twelve tips for teaching reflection at all levels of medical education. Med Teach.

[23] Tucker C, Efurd M, Turley S. e-Portfolios and self-assessment in dental hygiene education: a pilot study. J Allied Health. 2019;48(3):217–9 PMID: 31487361. 31487361

[24] Botelho M, Bhuyan SY (2021). Reflection before and after clinical practice-enhancing and broadening experience through self-, peer- and teacher-guided learning. Eur J Dent Educ.

[25] Woldt JL, Nenad MW (2021). Reflective writing in dental education to improve critical thinking and learning: a systematic review. J Dent Educ.

[26] Krause F, Ziebolz D, Rockenbauch K, Haak R, Schmalz G (2022). A video- and feedback-based approach to teaching communication skills in undergraduate clinical dental education: The student perspective. Eur J Dent Educ.

[27] Lowe M, Rappolt S, Jaglal S, Macdonald G (2007). The role of reflection in implementing learning from continuing education into practice. J Contin Educ Health Prof.

[28] Loka SR, Doshi D, Kulkarni S, Baldava P, Adepu S (2019). Effect of reflective thinking on academic performance among undergraduate dental students. J Educ Health Promot.

[29] Tendahl M, Gibbs RWJ (2008). Complementary perspectives on metaphor: cognitive linguistics and relevance theory. J Pragmat.

