# Supplementary material for: Comparison of PRISM and numeric scale for self-assessment of learning progress during a clinical course in undergraduate dental students
Source: BMC Med Educ. 2022 Dec 24;22:894. doi: 10.1186/s12909-022-03967-7 (PMC9789606; doi:10.1186/s12909-022-03967-7)

**Supplementary figures**

**Supplementary figure 1**: Perceived benefit of interview, instrument and the combination of both depending on group and time point. The y-axis shows the mean values of the respective issue.


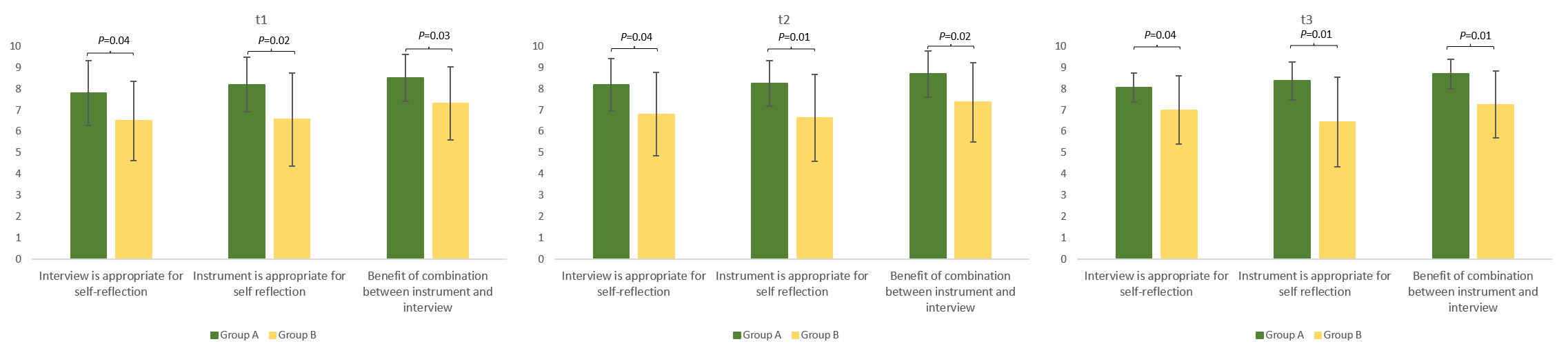

Supplement: Supplementary file 1 — Additional file 1: Supplementaryfigure 1. Perceived benefit of interview, instrument and the combination of both depending on group and time point. The y-axis shows the mean values of the respective issue. [file 12909_2022_3967_MOESM1_ESM.docx]
